# Face mask littering: environmental impacts, regulatory challenges, and AI-driven waste management strategies for the postpandemic era

**DOI:** 10.55730/1300-0144.6336

**Published:** 2026-04-28

**Authors:** Priyanka RATHEE, Sarita KHATKAR, Renu SEHRAWAT, Pooja RATHEE, Anurag KHATKAR, Esra KÜPELİ

**Affiliations:** 1 Geeta Institute of Pharmacy, Geeta University, Panipat, Haryana, India; 2 SGT College of Pharmacy, SGT University, Gurugram, Haryana, India; 3 Department of Pharmaceutical Sciences, Maharshi Dayanand University, Rohtak, Haryana, India; 4 Department of Pharmacognosy, Faculty of Pharmacy, Gazi University, Ankara, Turkiye

**Keywords:** COVID-19, environment, face mask, pollution, postpandemic, toxicity

## Abstract

The widespread use of polypropylene-based face masks during the COVID-19 pandemic created a significant and persistent source of plastic pollution. This review aims to provide an integrative assessment of the environmental, toxicological, and public health implications of face mask litter and evaluate current waste management challenges and emerging technological solutions in the postpandemic context. A narrative review approach was employed, synthesizing literature published between 2020 and 2025 and indexed in major scientific databases including Web of Science, Scopus, and PubMed. Studies were selected based on their relevance to the issues of environmental contamination, microplastic generation, toxicological effects, regulatory frameworks, and artificial intelligence (AI)-based waste management strategies. The findings indicate that discarded face masks are a major source of microplastics and hazardous chemical additives, contributing to environmental contamination across terrestrial, aquatic, and atmospheric systems. These materials may enter human exposure pathways through inhalation, ingestion, and dermal contact, with potential implications for respiratory health, systemic toxicity, and endocrine disruption. In addition, current waste management systems are shown to be insufficient for handling personal protective equipment-related waste, particularly under pandemic conditions. While AI-driven technologies can improve waste detection, sorting, and monitoring efficiency, their real-world scalability remains constrained by data limitations, infrastructure requirements, and environmental trade-offs. Overall, this review emphasizes that face mask pollution constitutes a multidimensional environmental and public health challenge requiring integrated responses. Strengthening regulatory frameworks, advancing sustainable material design, and combining AI-based tools with robust waste management systems are essential for mitigating long-term risks and improving preparedness for future global health crises.

## Graphical abstract

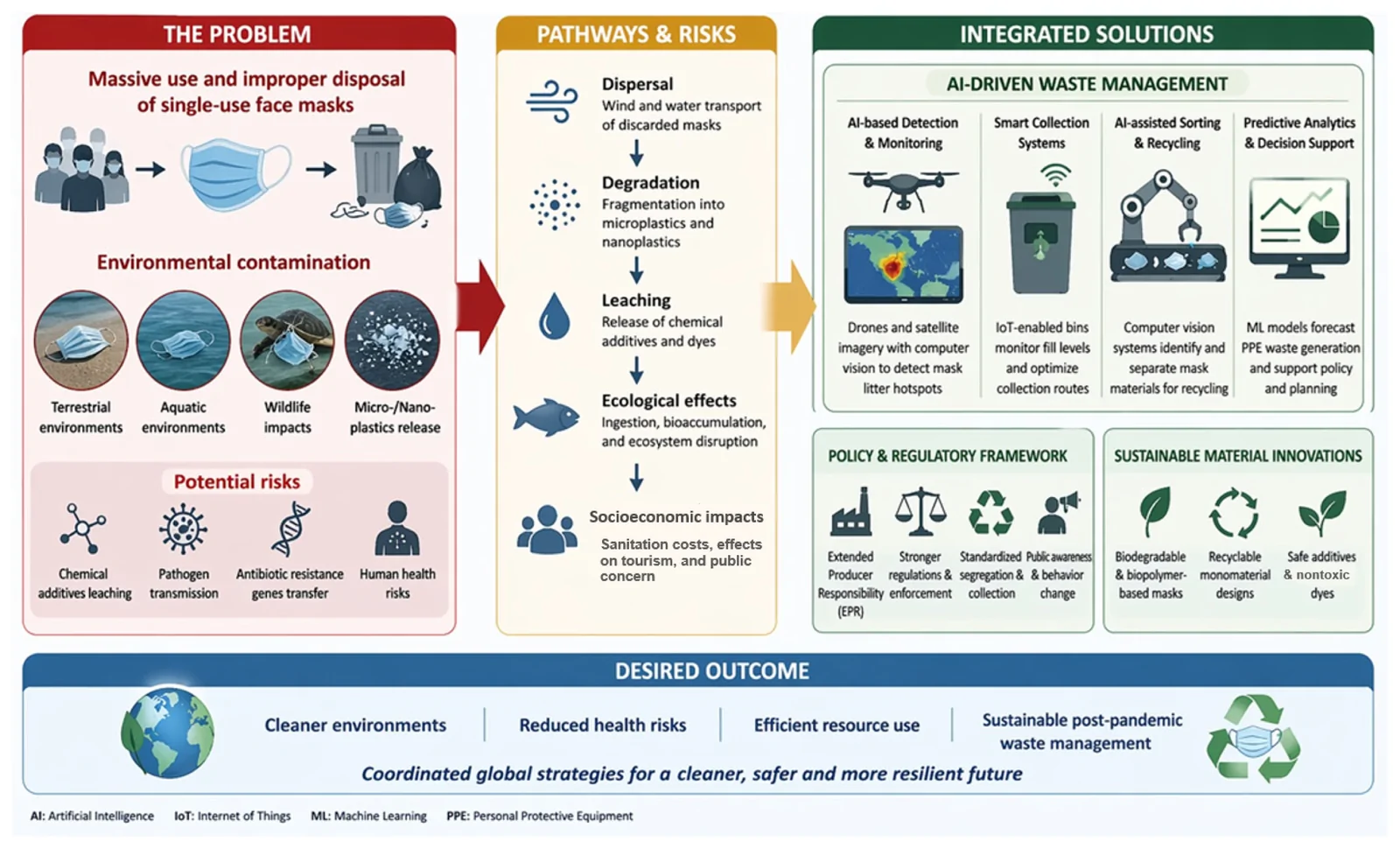

## 1. Introduction

The COVID-19 pandemic triggered an unprecedented global surge in the consumption of single-use plastics, particularly polypropylene (PP)-based personal protective equipment (PPE) such as face masks. As new SARS-CoV-2 variants with higher transmissibility continued to emerge, including the Omicron sublineages, widespread mask use became a central public health intervention and contributed to a substantial increase in plastic waste generation worldwide [1–3]. Face masks played a key role in reducing viral transmission and protecting communities [4–6], but their improper disposal led to the rapid accumulation of contaminated plastic debris in terrestrial and aquatic environments [7,8].

Disposable masks, primarily composed of PP microfibers, degrade slowly and release microplastics (MPs), nanoplastics (NPs), and chemical additives during environmental weathering [9,10]. These particles have been detected across diverse ecosystems, where they pose ecological hazards through ingestion, biofilm formation, pollutant adsorption, and the potential transfer of pathogens or antibiotic-resistance genes [10–13]. Several studies have also highlighted the risks associated with phthalates, dye residues, flame retardants, and other hazardous chemicals that leach from discarded face masks [14,15].

The rapid increase in mask waste during the pandemic exposed notable weaknesses within existing waste management systems. Many countries experienced insufficient waste segregation, limited recycling capacity, inadequate treatment infrastructure, and inconsistent enforcement of environmental regulations [16–18]. Public awareness and behavioral factors further contributed to improper disposal practices, exacerbating mask litter in streets, waterways, beaches, and urban spaces [19,20].

As societies transition into the postpandemic era, there is a growing need to understand the environmental fate and toxicological implications of discarded masks, strengthen regulatory frameworks, and develop sustainable alternatives such as biodegradable or biopolymer-based mask materials [21,22]. Technological innovations, particularly including artificial intelligence (AI), are discussed in later sections of this review as potential tools for improving waste detection, sorting, forecasting, and ensuring resource recovery within overburdened waste infrastructures [23–25].

Overall, the escalating environmental footprint of face mask litter highlights the urgent need for integrated approaches that combine environmental monitoring, public engagement, regulatory interventions, sustainable material development, and AI-driven waste management strategies. This review synthesizes current evidence on the environmental impacts, toxicological risks, and policy challenges associated with face mask litter and evaluates emerging technological solutions that could support sustainable waste practices in the postpandemic era.

**Figure 1:**
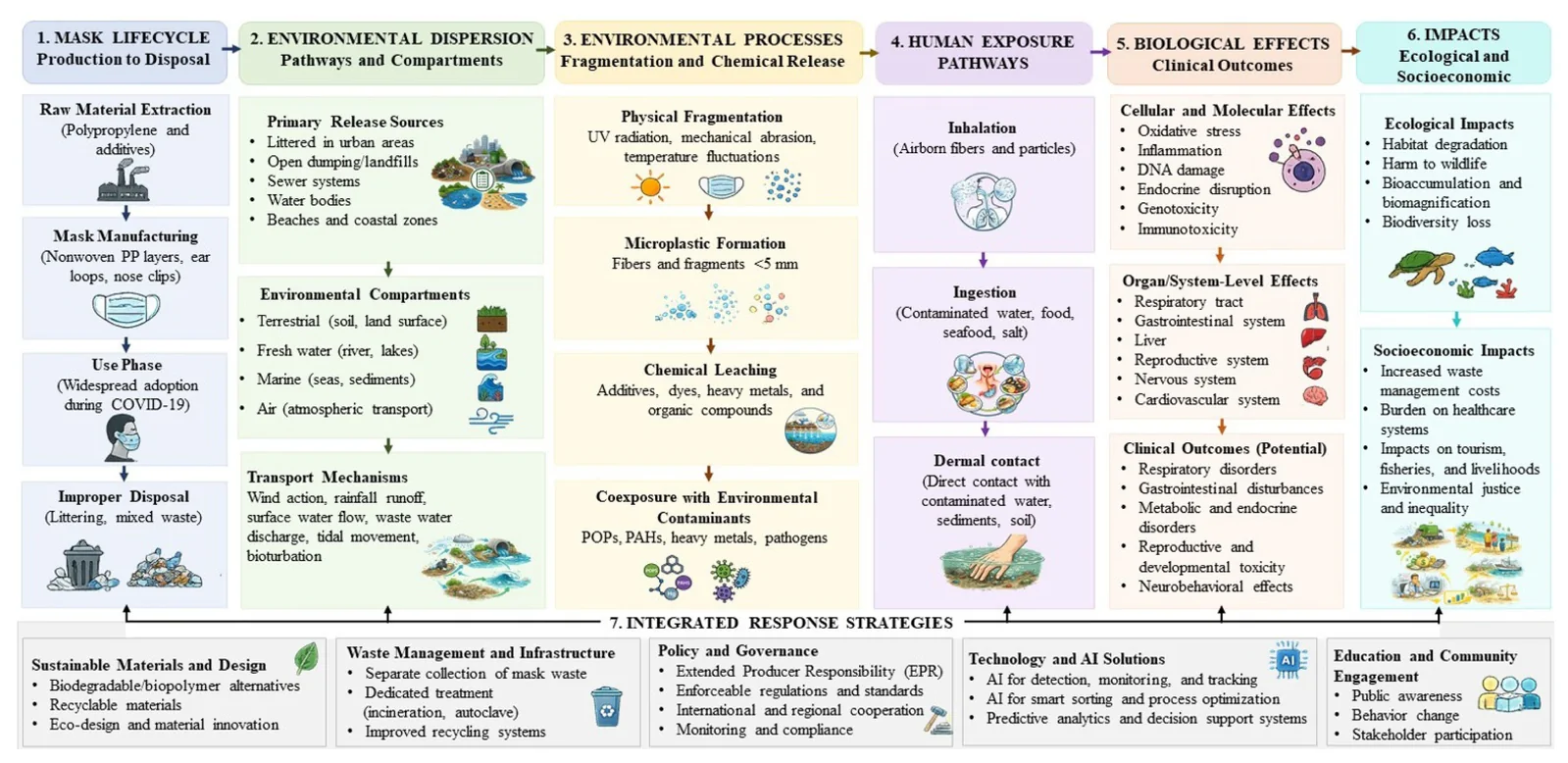
Integrated conceptual framework illustrating the lifecycle of face masks, environmental dispersion pathways, microplastic and chemical release, human exposure routes, associated biological and clinical effects, and corresponding response strategies in the postpandemic context.

Despite the growing body of literature on PPE-related plastic pollution and MPs, existing reviews have predominantly focused on isolated aspects such as environmental occurrence, degradation mechanisms, or toxicological effects. There remains a lack of integrative frameworks that systematically connect environmental fate, human and ecological risks, waste management limitations, regulatory responses, and emerging technological solutions within a unified postpandemic context (Figure 1).

In addition, several critical knowledge gaps remain, with a limited understanding of long-term and low-dose human exposure to mask-derived MPs and chemical additives, as well as insufficient real-world evidence on the scalability of emerging technological solutions such as AI-based waste management systems. Methodological inconsistencies further hinder cross-study comparability and standardized risk assessment.

This review provides an integrated perspective by linking environmental fate, toxicological risks, waste management challenges, and emerging technological solutions within a unified postpandemic framework. It also identifies key knowledge gaps and outlines a forward-looking research and policy agenda for sustainable waste management.

Importantly, beyond environmental consequences, face mask-derived MPs and associated chemical additives constitute an emerging concern for human health. Potential exposure pathways, including inhalation of airborne microfibers, ingestion through contaminated food and water, and dermal contact, raise questions regarding their clinical relevance. These exposures may contribute to respiratory irritation, systemic inflammation, and endocrine disruption, although current evidence remains limited and requires further investigation. In this context, understanding the intersection between environmental contamination and public health is essential for developing effective and clinically relevant mitigation strategies.

## 2. Methodology of the review

This study was designed as a narrative review based on a structured synthesis of peer-reviewed literature published between 2020 and 2025, with emphasis on high-impact journals and widely cited studies in the fields of MPs, waste management, and environmental health. A structured literature search was conducted using major scientific databases, including Web of Science, Scopus, and PubMed. Additional sources such as Google Scholar were used to identify more recent publications and relevant gray literature. The search process included combinations of keywords such as “face mask waste,” “COVID-19 PPE pollution,” “microplastics,” “waste management,” “biodegradable masks,” and “artificial intelligence in waste management.”

Studies published between 2020 and 2025 were selected to capture developments during and after the COVID-19 pandemic. Studies were included based on their relevance to environmental impacts, toxicological risks, waste management challenges, regulatory frameworks, and technological solutions. Priority was given to studies providing quantitative data, real-world observations, or integrative analyses, while widely cited and foundational studies were used to contextualize key concepts.

The selected literature was critically evaluated and thematically organized to provide an integrated perspective on the challenges and potential solutions related to face mask litter. Although this approach does not follow a formal systematic review protocol such as that advocated by the PRISMA guidelines, it ensures transparency, reproducibility, and comprehensive coverage of the available literature.

## 3. Increased mask consumption during the emergence of Omicron and other SARS-CoV-2 variants

The emergence of highly transmissible SARS-CoV-2 variants, including the Omicron lineage and its subvariants, significantly increased global reliance on face masks as a primary public health intervention. These variants demonstrated enhanced infectivity and strong immune-evasion characteristics, resulting in recurrent waves of community transmission even in vaccinated populations [1–3]. As a result, many countries reinforced mask mandates in public spaces, healthcare settings, workplaces, and transportation networks.

This period saw a substantial rise in the production, distribution, and use of single-use PP masks, further intensifying plastic waste generation [4,26]. Although mask-wearing proved effective in reducing viral spread and protecting individuals and communities [5,6], the associated increase in consumption contributed directly to the accumulation of discarded masks in the environment.

Thus, the emergence of Omicron and its subvariants amplified face mask pollution by prolonging mask mandates and driving higher global demand for disposable PPE.

## 4. Face masks: an effective tool for pandemic mitigation

Face masks served as one of the most effective and accessible nonpharmaceutical interventions during the COVID-19 pandemic, substantially reducing viral transmission and providing both individual and community-level protection. Their widespread global use, however, also increased plastic-based waste generation, highlighting the need to understand both their public health value and their environmental consequences [27,28].

### 4.1. Control of respiratory emissions: droplet and aerosol source reduction

Face masks function as a critical source-control intervention by substantially reducing the outward emission of respiratory droplets and aerosols generated during routine expiratory activities, including breathing, speaking, coughing, and sneezing. Respiratory particles span a continuum of aerodynamic diameters, from droplets of >100 μm that follow ballistic trajectories to aerosols of <5 μm capable of prolonged airborne suspension, each contributing to transmission through distinct physical pathways. Experimental and observational studies demonstrate that mask materials, whether woven or nonwoven, can significantly attenuate both the number and momentum of expelled particles, thereby reducing their capacity to disperse into the surrounding microenvironment [6,29].

**Figure 2:**
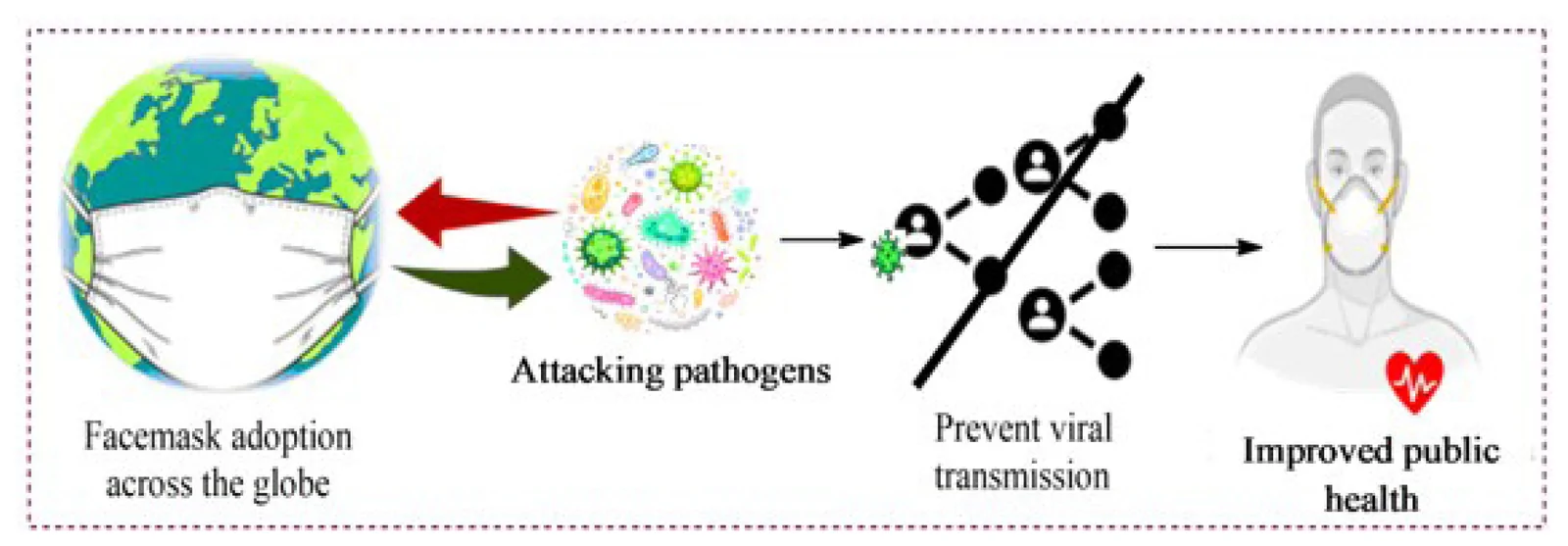
Protective role of face masks in reducing exposure to airborne pathogens and limiting transmission.

This mitigation effect is particularly consequential given the well-documented role of asymptomatic and presymptomatic carriers in sustaining transmission chains. Individuals lacking overt symptoms may emit infectious viral particles at concentrations comparable to symptomatic cases, leading to unrecognized yet epidemiologically significant spread. Masks act by intercepting respiratory emissions at their point of origin, decreasing forward particle propagation, limiting plume reach, and reducing aerosolization resulting from droplet fragmentation during exhalation (Figure 2).

Furthermore, by lowering the overall particle load within shared indoor spaces, consistent mask use disrupts transmission pathways across diverse settings, including households, occupational environments, schools, healthcare facilities, public transit, and other community venues. This preventive impact becomes increasingly critical under conditions of poor ventilation, high occupancy, or prolonged exposure, where aerosol accumulation can elevate the risk of airborne transmission. Collectively, these mechanisms position masks as an essential component of multilayered infection-control strategies aimed at reducing both individual exposure and population-level transmission dynamics [6,29].

### 4.2. Protection of the wearer

Masks also provide inward filtration, reducing the inhalation of airborne virus-containing particles. Although filtration capacity varies across mask types, evidence suggests that cloth masks, surgical masks, and respirators all provide measurable protection, especially in confined or poorly ventilated environments [5].

### 4.3. Integration with public health interventions

During the pandemic, mask use functioned synergistically with other public health measures including vaccination, distancing, and hand hygiene. Multiple studies have confirmed that layered interventions produce the greatest reduction in transmission, underscoring the role of masks as a central component of comprehensive public health strategies [30,31].

### 4.4. Social and behavioral dimensions of mask use

Mask adoption reflects broader behavioral and societal adaptation during health crises. Widespread mask-wearing contributed to social resilience, increased risk awareness, and collective responsibility toward protecting vulnerable populations. Community trust and compliance strongly influence the effectiveness of mask interventions [32].

## 5. Assessing the environmental, social, and public health impacts of face mask litter

The global escalation in face mask use during the COVID-19 pandemic unintentionally introduced a persistent source of plastic pollution. Improperly discarded masks contribute to environmental degradation, compromise public spaces, and create potential sanitation risks. These impacts span environmental, social, and public health dimensions, reflecting the complex and interconnected nature of mask-derived pollution. Understanding the multidimensional impacts of mask litter is essential for designing sustainable mitigation strategies [7].

Although the available literature consistently identifies discarded face masks as an emerging source of environmental contamination, the reported magnitude of this problem varies considerably across studies because of differences in sampling design, monitoring periods, environmental settings, and mask-density estimation methods. Urban hotspot surveys often report high visible litter loads, whereas broader landscape-level studies may underestimate the persistence and secondary fragmentation of mask-derived plastics. This methodological heterogeneity limits direct comparability across studies and complicates efforts to generate standardized estimates of environmental burden. In addition, while the environmental presence of mask litter is well documented, direct causal links between litter abundance and long-term ecosystem-level outcomes remain insufficiently quantified. This suggests that the current evidence base is stronger for documenting occurrence than for defining long-term ecological burden. Moreover, variations in environmental conditions, such as UV exposure, hydrodynamics, and mechanical stress, further influence degradation rates and MP release, contributing to inconsistent findings across studies. Future studies should prioritize harmonized monitoring protocols, longitudinal assessments, and cross-compartment tracking of mask-derived particles across soil, freshwater, marine, and atmospheric systems.

### 5.1. Environmental impact

Recent estimates indicate that the global consumption of face masks during the COVID-19 pandemic reached tens of billions of units per month, resulting in millions of tons of additional plastic waste annually. Even a small fraction of improperly discarded masks can contribute substantially to environmental contamination, with individual masks capable of releasing thousands of microfibers under environmental weathering conditions. Field-based assessments report variable densities of mask litter across different regions, with higher accumulation in densely populated urban areas, coastal zones, and regions with limited waste management infrastructure.

Despite increasing evidence of environmental contamination, the current data remain heterogeneous in terms of their strength. Most quantitative estimates are derived from localized field surveys or laboratory-based simulations, which may not fully capture real-world variability. Coastal and marine ecosystems appear to be among the most affected due to hydrodynamic transport and accumulation processes, while urban environments serve as primary sources of mask litter. However, long-term ecosystem-level impacts remain insufficiently quantified, highlighting the need for standardized monitoring approaches and large-scale comparative studies across different climatic and socioeconomic contexts.

**Figure 3:**
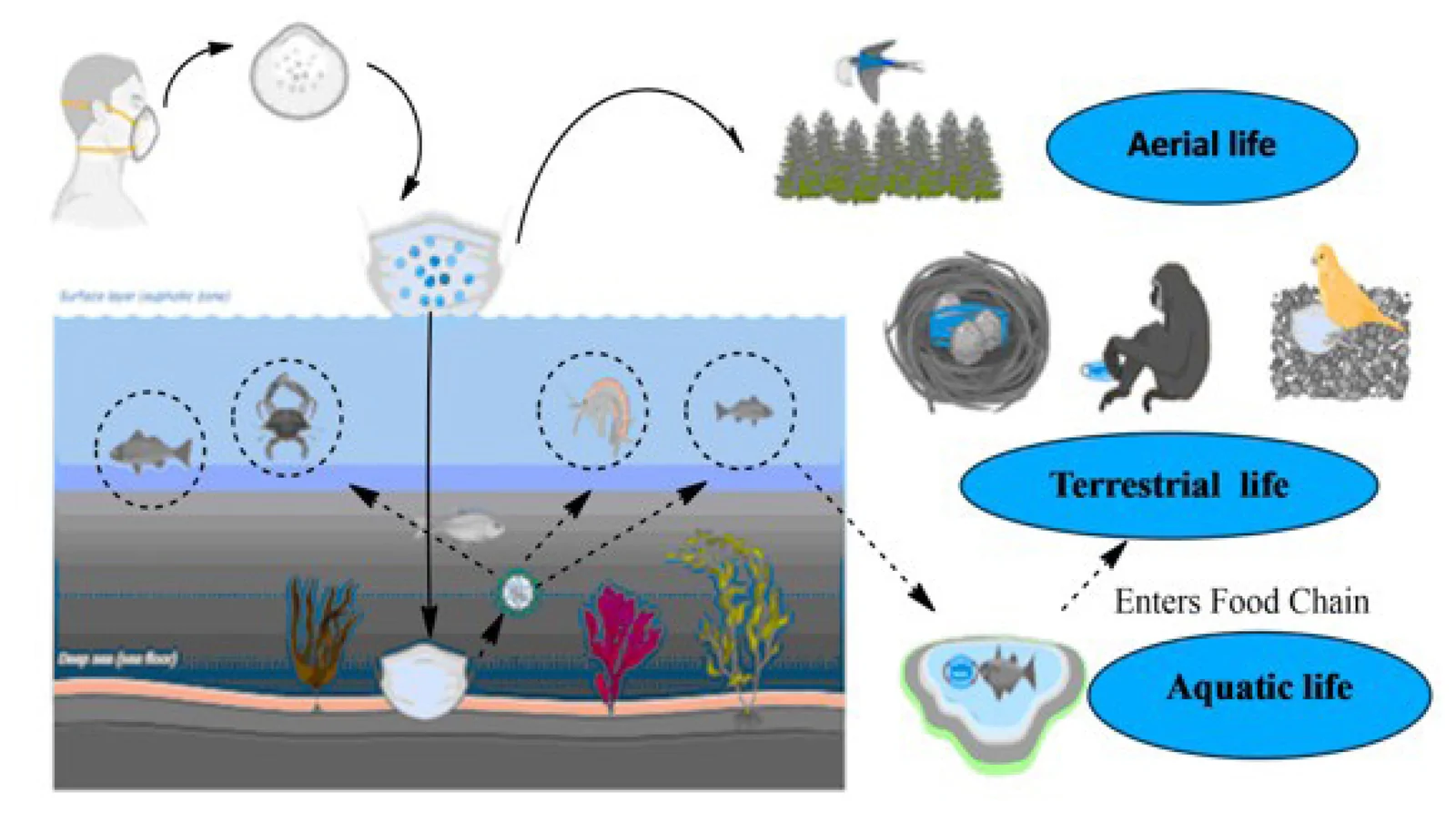
Environmental transport of fragmented face masks across soil, water, and atmospheric systems and their interactions with living organisms.

Under environmental conditions, discarded PP face masks undergo fragmentation through ultraviolet radiation, mechanical abrasion, and hydrothermal processes, resulting in the release of MPs and fibrous debris [9]. These MPs disperse through multiple pathways, including surface runoff, riverine transport, and wind-driven atmospheric movement, enabling long-range distribution across diverse ecosystems (Figure 3) [33,34].

Mask-derived plastic particles contribute to habitat deterioration by altering the soil structure, reducing aeration, and affecting water permeability. In aquatic systems, accumulated fibers increase turbidity and interfere with nutrient cycling. The polymer matrix of PP masks can also release chemical additives, such as dyes, stabilizers, and residual monomers, contributing to localized chemical pollution [14]. In addition, biofilm formation on mask fragments promotes microbial colonization and facilitates the transport of environmental pathogens, potentially influencing microbial community composition [11].

Improperly discarded masks further degrade the aesthetic quality of urban and natural landscapes, impacting tourism, public perception, and social well-being [19]. Accumulation of contaminated masks in public spaces may also increase sanitation challenges and contribute indirectly to public health concerns [35]. Overall, mask litter acts as a persistent environmental pollutant with multicompartmental transport potential and long-term ecological implications [8,10], although the magnitude of these impacts remains subject to ongoing scientific uncertainty.

### 5.2. Social impact

Face mask litter exerts notable social consequences by degrading the aesthetic quality of public spaces and influencing community perceptions of environmental responsibility. The presence of discarded masks in urban environments, recreational areas, and waterways diminishes the visual cleanliness of shared spaces and contributes to negative public attitudes regarding civic behavior [19]. Persistent visible litter may weaken social cohesion by fostering frustration, blame, or stigmatization among community groups, particularly where waste mismanagement is interpreted as carelessness or disregard for communal well-being. Moreover, declining environmental quality in neighborhoods can impact the overall quality of life and reduce public engagement in proenvironmental actions, reinforcing a cycle of littering and neglect.

### 5.3. Public health impact

**Figure 4:**
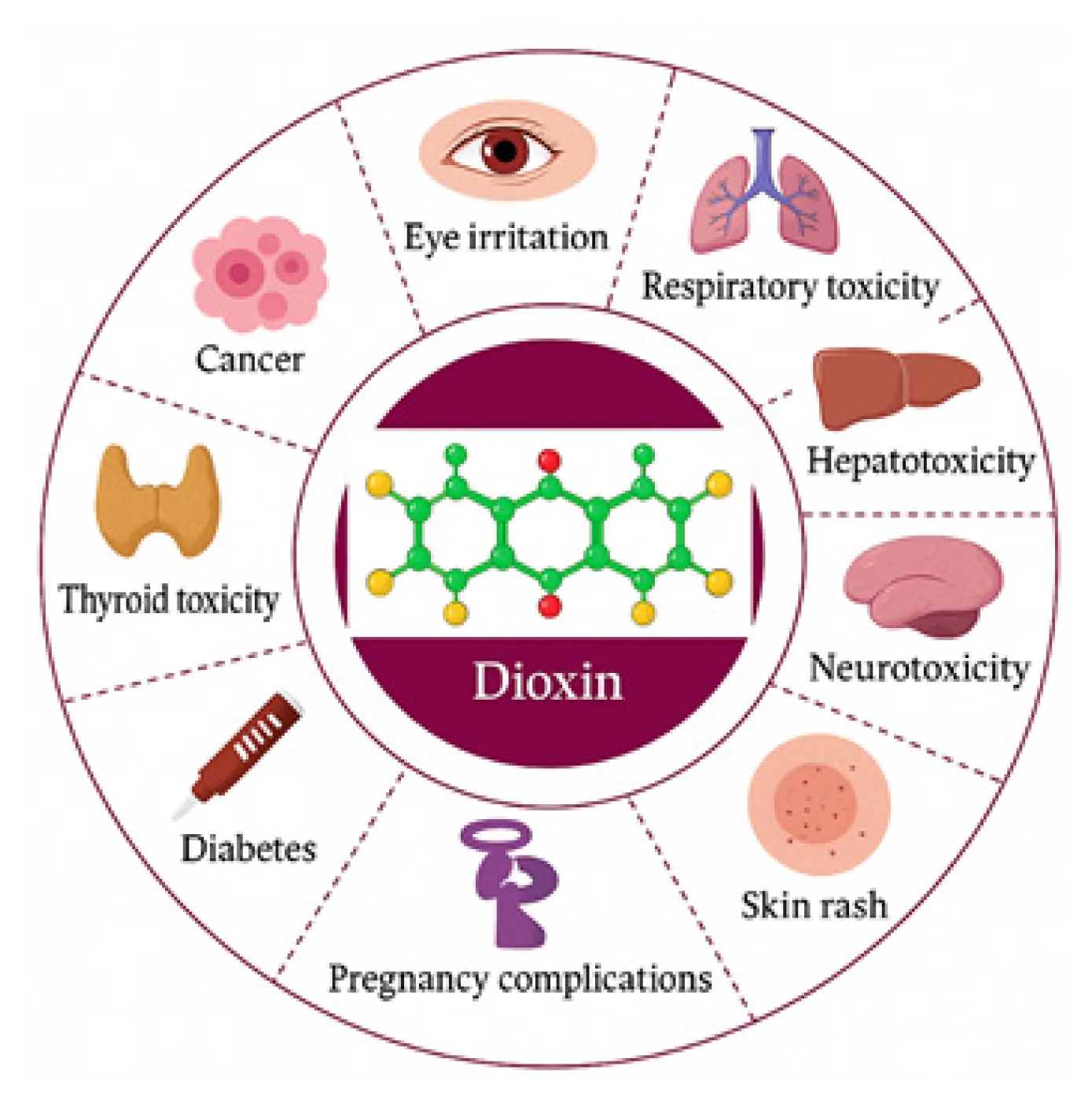
Public health risks associated with improper disposal of face masks and the resulting environmental contamination.

From a public health standpoint, improperly discarded face masks pose significant sanitation and contamination risks. Masks used in community and healthcare settings may retain viral particles, bacteria, or other pathogens, creating potential exposure hazards when left in densely populated or high-traffic areas [35]. Waste-handling personnel and sanitation workers are particularly vulnerable due to direct contact with contaminated PPE, especially in the absence of dedicated collection systems. Accumulated mask waste can compromise hygiene standards, interfere with sanitation practices, and undermine broader infectious disease prevention efforts, potentially contributing to localized transmission risks and reduced public confidence in municipal waste management systems (Figure 4).

Beyond immediate contamination concerns, emerging evidence suggests that chronic exposure to MPs and mask-derived chemical additives may have clinically relevant implications. Inhaled microfibers may enter the respiratory tract and contribute to inflammatory responses, while ingestion through contaminated food and water may result in systemic exposure. Furthermore, additives such as phthalates and flame retardants have been associated with endocrine disruption, reproductive toxicity, and metabolic disorders. However, current evidence remains limited, and the translation of these findings to real-world human health outcomes requires further investigation. These observations highlight the need to consider face mask pollution not only as an environmental issue but also as a potential contributor to long-term public health burden.

Occupational exposure is an additional critical dimension of risk. Healthcare workers, sanitation personnel, and waste management staff are frequently exposed to improperly discarded or contaminated masks, increasing the likelihood of contact with biological pathogens and hazardous residues. Repeated exposure under insufficient protective conditions may elevate the risk of respiratory, dermal, and infectious complications, particularly in settings lacking standardized safety protocols. This underscores the importance of targeted occupational protection measures and structured waste handling systems.

### 5.4. Assessment strategies

A comprehensive and interdisciplinary assessment framework is essential for evaluating the multifaceted environmental, social, and public health impacts associated with mask litter. Effective evaluation should integrate ecological risk assessment, environmental monitoring, social perception surveys, and public health surveillance to capture the full extent of mask-derived pollution [35,36]. Policy analysis is equally critical to identify regulatory gaps and guide the development of targeted interventions. Collaboration among governmental agencies, local authorities, community organizations, researchers, and private stakeholders is necessary to improve waste segregation, promote responsible disposal behaviors, and strengthen environmental resilience. Key assessment strategies and corresponding methodological approaches are summarized in Table 1.

## 6. Toxicological impacts of PP-based littered face masks

Mask-derived MPs and NPs pose significant toxicological risks to a wide range of organisms. Studies report alterations in feeding behavior, reduced growth, impaired reproduction, and changes in enzymatic activity in both plants and animals following exposure to PP, PE, PS, PAN, and other polymeric MPs [40–46]. Notably, the severity and nature of these effects vary across polymer types and exposure conditions, reflecting differences in physicochemical properties such as particle size, surface area, and degree of environmental weathering. These effects arise from both the physical abrasion caused by particles and the chemical toxicity associated with leached additives. The reported toxicological effects of the major polymers present in face masks are summarized in Table 2.

The ability of MPs to adsorb persistent organic pollutants, dyes, heavy metals, and endocrine-disrupting chemicals further enhances their toxic potential [12,14]. Biofilm accumulation on MPs may facilitate the transport of pathogenic microorganisms and antibiotic-resistance genes, influencing microbial dynamics in aquatic ecosystems [11]. The trophic transfer of MPs across food webs introduces additional ecological risks and constitutes a possible exposure pathway for humans via seafood, drinking water, and airborne particles [13]. However, the extent to which these pathways contribute to measurable human health outcomes remains insufficiently quantified, highlighting a critical gap between environmental detection and health risk assessment.

Chemical additives in face masks, including phthalates, nonylphenol, triclosan, organotin compounds, and polybrominated diphenyl ethers, exhibit diverse toxicological profiles. These compounds are linked to endocrine disruption, reproductive toxicity, neurotoxicity, immunotoxicity, and metabolic disorders [48–54]. Importantly, the combined effects of these additives may result in mixture toxicity, which is often not adequately captured in single-compound experimental studies. Phthalates detected in mask samples worldwide highlight the potential for additive leaching and human exposure [15]. A concise overview of the toxicological effects associated with these mask-related chemical additives is provided in Table 3.

Overall, mask-derived MPs and associated chemicals present significant toxicological risks, underscoring the importance of sustained environmental monitoring, policy interventions, and safer material design. Table 4 provides a summary of key mitigation strategies aimed at reducing these biological and chemical hazards [55–59]. Despite this growing body of evidence, most studies remain limited to short-term assessments and controlled experimental conditions, which may not fully capture the complexity of real-world exposure scenarios. Collectively, these findings highlight the urgent need for proactive mitigation strategies, as the continued environmental accumulation of mask-derived MPs and hazardous additives may lead to long-term cumulative toxicological effects across ecosystems and human populations.

The toxicological literature suggests that mask-derived MPs and associated additives may pose significant risks to biota and potentially to human health; however, important limitations to this knowledge remain. Much of the available evidence is derived from laboratory-based exposure models using controlled particle concentrations that may not fully reflect environmentally realistic conditions. In addition, toxicological findings vary depending on polymer type, particle size, weathering status, additive composition, and the test organism used, making cross-study comparisons difficult. Another unresolved question is the relative contribution of physical particle stress versus chemical toxicity from leached additives. Thus, while the evidence is sufficiently strong to justify precautionary concern, major knowledge gaps persist regarding chronic low-dose exposure, mixture toxicity, trophic transfer under natural conditions, and cumulative human health impacts. Future research should prioritize environmentally relevant exposure scenarios, standardized testing protocols, and interdisciplinary approaches linking ecotoxicology with human health risk assessment.

While a substantial body of toxicological evidence demonstrates the adverse effects of mask-derived MPs and associated chemical additives, it is important to distinguish between experimental findings and real-world exposure scenarios. Most current studies are based on controlled laboratory conditions using relatively high particle concentrations, which may not fully reflect environmentally relevant exposure levels. Consequently, the direct translation of these findings to human health outcomes remains uncertain.

In real-world contexts, human exposure is likely to occur through multiple pathways, including the inhalation of airborne microfibers, ingestion via contaminated food and water, and dermal contact with mask materials. Inhaled particles may be deposited along the respiratory tract and may potentially contribute to airway irritation and inflammatory responses, while ingested particles may interact with gastrointestinal systems and undergo systemic distribution. Moreover, chemical additives such as phthalates and flame retardants are known to be associated with endocrine disruption, reproductive toxicity, and metabolic disorders.

Taken together, these findings suggest that mask-derived contaminants constitute a plausible, though not yet fully quantified, risk to human health. Bridging the gap between experimental toxicology and epidemiological evidence will be essential for accurately assessing the long-term clinical implications.

## 7. Navigating challenges associated with current waste management practices

Effective waste management is essential for minimizing environmental degradation and protecting public health. However, the rapid increase in pandemic-related waste, and particularly single-use plastics such as face masks, has exposed several structural weaknesses within existing waste management systems. These challenges include inadequate segregation practices, limited recycling capacity for contaminated or mixed-material waste streams, and insufficient infrastructure to handle sudden surges in both medical and household waste. Importantly, these weaknesses are not new, but they were amplified under the exceptional conditions of the pandemic, revealing preexisting systemic fragilities. In many regions, overwhelmed waste facilities struggled to maintain operational efficiency, revealing longstanding vulnerabilities in collection, transport, and treatment processes. Moreover, gaps in regulatory enforcement, inconsistent implementation of waste protocols, and limited public awareness further complicate the safe disposal and treatment of high-volume plastic waste generated during health emergencies. In addition, the heterogeneous composition of PPE waste, often combining multiple polymer layers and potential biological contamination, further limits conventional recycling pathways. Together, these limitations underscore the need for more resilient, adaptable, and sustainable waste management frameworks capable of responding effectively to future crises and public health emergencies.

The literature broadly agrees that existing waste management systems were not designed to accommodate the sudden increase in PPE-related plastic waste during the pandemic; however, the severity of the failures differed substantially across regions. Studies from low- and middle-income settings tend to emphasize infrastructure shortages and informal disposal pathways, whereas reports from high-income countries more often highlight issues related to segregation, contamination, and the limited recyclability of mixed-material waste. This indicates that the problem is not uniform; it is context-dependent. These regional disparities highlight the importance of tailoring waste management interventions to local socioeconomic and institutional conditions rather than adopting uniform, one-size-fits-all approaches. A major limitation in the current evidence is the lack of comparative assessments of which interventions are most effective under different regulatory and economic conditions. More region-specific implementation studies are needed to determine whether investments should prioritize collection systems, treatment infrastructure, producer responsibility mechanisms, or public behavior change strategies. Furthermore, longitudinal studies assessing the long-term performance and resilience of these interventions remain scarce.

Real-world experiences during the COVID-19 pandemic demonstrated significant variation in waste management performance across regions. High-income countries generally maintained structured collection systems and implemented temporary regulatory adaptations for medical and PPE waste, although challenges related to segregation and recyclability persisted. In contrast, many low- and middle-income countries faced substantial constraints, including limited infrastructure, informal disposal practices, and inadequate regulatory enforcement, leading to increased environmental leakage of mask waste. Comparative analyses suggest that regulatory effectiveness is closely linked to institutional capacity, public compliance, and the availability of dedicated waste treatment facilities, with higher performance consistently observed in systems with integrated governance and enforcement mechanisms. These differences highlight that waste management strategies must be context-specific rather than universally applied.

Despite the availability of advanced waste management technologies and policy frameworks, their practical implementation remains constrained by economic and operational factors. High costs associated with specialized collection systems, treatment facilities, and advanced recycling technologies can limit adoption, particularly in resource-constrained settings. In addition, behavioral factors such as low public awareness, improper segregation practices, and resistance to regulatory compliance further hinder effective implementation. Logistical challenges, including transportation, workforce capacity, and monitoring systems, also affect system performance. Therefore, improving waste management outcomes requires not only technological innovation but also sustained financial investment, institutional strengthening, and community-level engagement to ensure long-term feasibility, scalability, and real-world applicability.

### 7.1. Inadequate infrastructure and resources

Many regions lack sufficient infrastructure to manage rising waste volumes, with limited recycling capacity, insufficient treatment facilities, and constrained landfill availability. These limitations are especially pronounced in low- and middle-income countries, where inadequate financial investment further restricts the development and maintenance of waste management systems [16]. Addressing this gap requires targeted investment in waste treatment technologies, improved collection systems, and capacity-building initiatives.

### 7.2. Complex regulatory frameworks

Waste management regulations often vary significantly across jurisdictions, resulting in inconsistent waste classification, disposal standards, and recycling targets. Fragmented regulatory landscapes complicate compliance and hinder effective environmental oversight [18]. Harmonizing regulatory frameworks and improving cross-sectoral coordination can enhance operational efficiency and enforcement.

### 7.3. Emerging environmental hazards

Urbanization, industrial expansion, and changing consumption patterns have introduced new environmental risks, including plastic pollution, hazardous chemicals, and electronic waste. Improper handling or disposal of these materials may lead to chemical leaching, heavy metal contamination, and long-term ecological damage. Strategies such as extended producer responsibility (EPR), sustainable product design, and advanced waste treatment technologies are essential for mitigating these risks [60].

### 7.4. Public awareness and behavior barriers

Public participation plays a crucial role in waste reduction and proper disposal. However, behavioral barriers, including low awareness, misinformation, and cultural attitudes, limit the effectiveness of waste management initiatives. Community engagement, targeted educational programs, and incentive-based recycling schemes can help promote sustainable disposal practices [61].

### 7.5. Climate change and system resilience

Climate change introduces additional challenges for waste management infrastructure due to rising sea levels, extreme weather events, and disruptions to collection and disposal systems. These impacts may exacerbate environmental contamination and threaten public health. Strengthening system resilience through infrastructure upgrades, emergency preparedness, and climate-adaptive planning is necessary to ensure the continuity of waste services under changing climatic conditions [18].

## 8. Proposed solutions for a more sustainable postpandemic world

The surge in single-use plastic face masks during the COVID-19 pandemic underscored the need for sustainable materials, improved waste valorization strategies, and more resilient waste management systems. Postpandemic sustainability efforts require integrative approaches that simultaneously reduce environmental burdens, support circular economy principles, and strengthen public health preparedness.

### 8.1. Biopolymer-based alternatives for sustainable face masks

Biopolymer-based face masks have emerged as promising substitutes for conventional PP masks due to their biodegradability, lower carbon footprint, and reliance on renewable feed stocks [21]. Polymers such as polylactic acid (PLA), polyhydroxyalkanoates (PHA), starch derivatives, chitosan, and cellulose-based materials can degrade into environmentally benign compounds under appropriate composting or natural conditions [22,62].

Advances in material engineering, including solvent-free processing, melt extrusion, and 3D printing, enable improved mechanical strength, filtration efficiency, and antimicrobial properties in biopolymer masks while reducing production-related energy use [63]. Table 5 summarizes recent developments in biodegradable mask technologies.

### 8.2. Role of polyphenols in circular waste management systems

Polyphenols, abundant in plant-derived materials, facilitate multifunctional applications in sustainable waste management strategies. When incorporated into biopolymer matrices, polyphenol-rich extracts enhance the mechanical performance, barrier properties, and biodegradability of ecofriendly packaging and protective materials [69]. Their strong adsorption and chelating capacities enable the removal of dyes, metals, and organic pollutants from wastewater systems, supporting greener treatment processes [70]. In soil remediation, polyphenol-rich organic amendments can improve nutrient cycling and microbial stability [71]. Collectively, these properties position polyphenols as valuable components in circular waste management frameworks [72].

Polyphenols also serve as natural preservatives with broad applications in the food, cosmetic, and pharmaceutical sectors, and their integration into biodegradable systems supports safer and more environmentally responsible material development [73,74].

### 8.3. Polymer recycling approaches for postpandemic waste

Polymer recycling is essential for recovering value from discarded masks and reducing dependence on virgin plastics.

#### 8.3.1. Mechanical recycling

Mechanical recycling involves shredding, washing, and reprocessing PP or polyethylene mask components into pellets that can be reused in manufacturing sectors such as packaging or construction materials [8]. This method is cost-effective and scalable but requires efficient waste segregation systems.

#### 8.3.2. Chemical recycling

Chemical recycling depolymerizes plastics into monomers or chemical feedstocks through processes such as pyrolysis, gasification, and chemolysis, enabling high-quality material recovery [56,75]. As summarized in Table 6, chemical recycling provides opportunities for upcycling mask waste into fuels and raw materials, supporting circular production loops.

#### 8.3.3. Biodegradation and composting

Biodegradable masks made from PLA, PHA, or starch-based polymers can be processed in industrial composting or anaerobic digestion facilities, generating compost or biogas while minimizing plastic persistence in the environment [76].

#### 8.3.4. Upcycling and repurposing

Innovative upcycling strategies, such as melt blending or compression molding, enable the transformation of mask waste into higher-value products, contributing to resource recovery and landfill diversion [77].

### 8.4. Integrated waste management systems

Integrated waste management frameworks combine prevention, recycling, recovery, and safe disposal within a unified system aligned with the waste hierarchy and circular economy principles [83]. Effective implementation requires coordinated governance, reliable waste segregation, improved collection and sorting systems, and context-specific technological solutions. Furthermore, the integration of advanced technologies and stakeholder collaboration has been highlighted as critical for improving system efficiency and sustainability in recent frameworks. Integrating polymer recycling, waste valorization, and sustainable material innovation can significantly reduce the long-term environmental footprint of pandemic-related plastic waste.

## 9. Global efforts to manage hazardous waste, including littered face masks

The COVID-19 pandemic substantially increased the generation of hazardous plastic waste, including discarded face masks. Effective governance frameworks are therefore essential to prevent environmental leakage, minimize public health risks, and support sustainable waste management systems. Global, regional, and national regulatory instruments collectively shape how hazardous waste is classified, transported, treated, and disposed of.

International agreements provide the foundation for global oversight. The Basel Convention of 1989 regulated transboundary movements of hazardous waste and prohibited exports to countries lacking adequate treatment capacity. The London Convention of 1972 restricted ocean dumping of hazardous materials, while the United Nations Convention on the Law of the Sea established broad obligations for protecting the marine environment and preventing land-based pollution inputs. Regional sea conventions have further complemented these frameworks by addressing marine litter and pollution control within specific geographic areas [84]. In addition, several countries have adopted national regulations governing the generation, storage, transportation, and disposal of hazardous waste [85]; examples from India are summarized in Table 7.

Despite the existence of robust legal frameworks, challenges persist due to limited enforcement capacity, insufficient infrastructure, and low stakeholder awareness. Notably, the effectiveness of these frameworks varies significantly across regions, reflecting differences in institutional capacity, economic resources, and regulatory priorities. Addressing these gaps requires targeted legislative measures specifically adapted to pandemic-related waste streams such as face masks. These differences are particularly evident when comparing high-income countries, which generally maintain more structured waste management and enforcement systems, with low- and middle-income countries where regulatory implementation and infrastructure capacity remain more limited.

Although multiple regulatory frameworks can be applied to hazardous or plastic waste streams, the literature indicates that the central challenge lies less in the absence of legal instruments than in inconsistent implementation, weak enforcement, and limited adaptation of existing rules to pandemic-specific waste streams such as discarded face masks. In this respect, there is a recurring gap between formal policy design and practical waste governance. This discrepancy highlights the need for context-specific implementation strategies rather than one-size-fits-all regulatory approaches. Another unresolved issue concerns whether used face masks should be universally classified as hazardous waste or managed through risk-based differentiation according to the setting and contamination status. Studies to date present differing perspectives on this issue, reflecting ongoing regulatory and scientific uncertainty. This regulatory ambiguity has important implications for cost, infrastructure, and disposal practices. Future policy research should therefore focus on implementation effectiveness, regulatory harmonization, and the development of operational criteria for PPE-specific waste classification, particularly under real-world and resource-constrained conditions.

### 9.1. Definition and categorization of hazardous waste

Clear classification systems are essential for consistent regulatory enforcement and for guiding waste handlers in selecting appropriate management pathways. Face masks composed of synthetic polymers, containing chemical additives, or contaminated with biological materials should be formally designated as hazardous waste to ensure that they are collected, stored, transported, and treated under controlled conditions. Such classification aligns disposal practices with national and international guidelines, minimizes risks to sanitation workers, and prevents the inadvertent release of infectious or chemically harmful components into the environment [90]. Establishing these clear categories also supports the development of standardized procedures and enhances compliance across diverse institutional and municipal waste management settings.

### 9.2. Governance of face mask manufacturing and labeling

Regulations should mandate transparent disclosure of mask composition, including polymer types and chemical additives. Standardized labeling requirements can guide end-users by providing instructions for safe disposal and information regarding environmental risks. Legislative frameworks may also incentivize or require the use of sustainable materials and low-impact manufacturing practices to reduce lifecycle environmental burdens [36].

### 9.3. Extended producer responsibility (EPR) schemes

EPR programs assign manufacturers responsibility for the postconsumer phase of their products. In the context of face masks, EPR can support collection, take-back systems, recycling initiatives, and environmentally sound disposal. By shifting part of the management burden from municipalities to producers, EPR schemes encourage companies to design masks with reduced environmental impact, improved recyclability, and lower material complexity. Financial incentives and compliance mechanisms help ensure participation and promote the integration of sustainable design principles throughout the product life cycle, ultimately contributing to more efficient and circular waste management systems [91].

### 9.4. Infrastructure for collection and disposal

Legislation should support the development of adequate treatment facilities and specialized collection systems for hazardous mask waste. Dedicated disposal bins in healthcare settings, public spaces, and workplaces, along with expanded decontamination and sterilization capacity, are necessary to prevent environmental release and reduce occupational hazards [16].

### 9.5. Public awareness and education

Regulatory frameworks should be complemented by targeted education campaigns that communicate proper handling and disposal practices for hazardous mask waste. Effective outreach across households, healthcare workers, businesses, and institutions can improve compliance and reduce environmental contamination [39].

Overall, comprehensive legislative action integrating classification standards, producer responsibility, sustainable manufacturing requirements, adequate infrastructure, and public education is essential for mitigating the environmental and public health risks associated with discarded face masks in the postpandemic era.

## 10. Technological innovations in AI-based waste management strategies

AI-based waste management strategies encompass a range of computational approaches, including machine learning, deep learning, computer vision, and data-driven decision-support systems. In the context of face mask and PPE-related waste, these methods can support object detection, material classification, contamination recognition, route optimization, and waste-generation forecasting. Among these, convolutional neural networks (CNNs) and other deep learning architectures are particularly relevant for image-based identification of discarded masks in streets, public spaces, beaches, and waste-sorting facilities, whereas supervised machine learning models are more commonly applied to predictive analytics for waste generation and collection planning. Transfer learning and advanced object-detection frameworks are increasingly used to address the scarcity of labeled PPE-specific datasets and improve model robustness.

Given the unique properties of PPE waste, including lightweight structure, high dispersibility, and potential contamination, AI-based systems can provide targeted solutions for detection, classification, and management as outlined in Section 5. AI-driven approaches, particularly those based on machine learning, deep learning, and computer vision, enable the identification, tracking, and management of mask litter across diverse environmental compartments. These systems support real-time environmental monitoring, improve sorting accuracy in recycling facilities, and optimize PPE-specific waste collection strategies. Emerging pilot-scale applications, including camera-based monitoring systems and smart waste facilities, demonstrate the potential of AI to enhance detection accuracy and operational efficiency, although large-scale implementation remains limited. Most existing studies remain at pilot or experimental stages, indicating a gap between technical feasibility and real-world deployment in municipal waste systems [92].

AI-based waste management strategies are increasingly presented as promising tools for improving detection, sorting, and planning; however, the current evidence remains uneven. Most studies demonstrate technical feasibility under controlled conditions, whereas robust validation in real-world municipal systems, particularly for PPE-specific waste, remains limited. Moreover, AI performance is highly dependent on dataset quality, local infrastructure, sensor availability, and institutional capacity, which limits generalizability across regions. In particular, the limited availability of labeled datasets specific to PPE waste remains a key bottleneck for the development and deployment of accurate AI models. Additional challenges include potential algorithmic bias arising from region-specific datasets and reduced model performance under variable environmental conditions such as occlusion, contamination, or weathering of mask materials. In addition, the environmental and economic costs associated with digital infrastructure, computational demand, and electronic waste introduce important sustainability trade-offs that must be considered.

Overall, AI should be regarded as an enabling component rather than a standalone solution, requiring integration with policy frameworks, infrastructure development, and behavioral interventions to achieve meaningful impact. Future research should therefore focus on improving dataset availability, strengthening real-world validation, and evaluating cost-effectiveness, scalability, and environmental trade-offs across diverse waste management contexts.

To facilitate the comparison of different AI approaches and their applicability to face mask waste management, a structured overview of commonly used methods, including their applications, strengths, and limitations, is provided in Table 8.

### 10.1. AI-driven waste sorting and recycling

AI-enabled computer vision systems can automatically classify waste materials by analyzing images captured from optical sensors or cameras. Machine learning models identify material types, separate recyclables from mixed waste streams, and enhance sorting accuracy [25]. Deep learning architectures further support the extraction of valuable materials by learning complex patterns and improving recognition performance under variable lighting, contamination, or morphological conditions. These systems not only reduce landfill burden and improve recycling efficiency but also enable real-time decision-making within automated sorting lines. Compared to conventional sorting methods, AI-assisted systems offer higher classification precision and adaptability to heterogeneous and contaminated waste streams, which is particularly relevant for PPE-related waste. Overall, AI-driven waste-sorting technologies strengthen resource recovery processes and support broader circular economy objectives by increasing material purity and operational efficiency.

For example, computer vision-based systems can be trained to specifically detect discarded face masks in streets, beaches, and water bodies using image recognition models. These systems can be integrated with surveillance cameras or drones to monitor mask litter density and spatial distribution in real time. In waste treatment facilities, AI-assisted sorting systems can distinguish mask materials (e.g., PP layers) from other plastic waste, improving recycling efficiency and reducing contamination risks. In addition, object-detection models (e.g., region-based and single-shot detectors) enable real-time identification of mask litter in complex and dynamic environments. However, the effectiveness of such systems may be constrained by variations in mask appearance due to environmental degradation, partial fragmentation, or occlusion in complex backgrounds. Furthermore, predictive models can estimate PPE waste generation based on epidemiological trends, population density, and policy measures, enabling more adaptive and responsive waste management strategies. Despite these advances, large-scale implementation remains limited and further validation under real-world operational conditions is needed to assess reliability, cost-effectiveness, and scalability.

In practice, CNN-based architectures are often integrated with transfer learning strategies to address the scarcity of labeled PPE-specific datasets, allowing models pretrained on general object-recognition tasks to be adapted to mask detection with relatively limited additional data. In automated sorting settings, AI systems can be integrated with optical sensors, robotic sorting units, and conveyor-based recognition platforms to improve material separation efficiency and reduce the manual handling of potentially contaminated waste. Despite these technological advances, however, system performance remains sensitive to dataset bias, environmental variability, and infrastructure constraints, which must be addressed to enable reliable deployment at scale.

### 10.2. Predictive analytics for waste production and collection

Machine learning algorithms can model temporal and spatial variations in waste generation by analyzing historical waste patterns, demographic variables, and environmental factors. Commonly used approaches include time-series models, regression-based machine learning algorithms, and ensemble methods that improve predictive accuracy under complex and dynamic conditions. Predictive analytics supports optimized waste collection schedules, improved fleet management, and cost-effective allocation of resources. Real-time AI monitoring can dynamically adjust collection routes, thereby lowering operational emissions and enhancing the responsiveness of municipal waste services [93].

Emerging pilot-scale applications suggest that AI can improve operational efficiency in waste monitoring and sorting, particularly when combined with camera-based surveillance, drone imaging, or smart-facility infrastructure. For example, computer vision systems have been explored for litter detection in urban and coastal environments, while predictive machine learning tools have been used to model fluctuations in waste generation and optimize collection schedules. However, most existing applications remain limited to pilot studies, simulation settings, or non-PPE-specific waste streams, indicating that large-scale validation for face mask waste management is still insufficient.

Despite their promise, AI-based waste management systems face important methodological and practical limitations. Model performance depends heavily on the quality, diversity, and representativeness of training datasets; in the case of face mask waste, the limited availability of labeled, PPE-specific datasets remains a major constraint. In addition, algorithmic bias may arise when models are trained on narrow environmental conditions or region-specific image sets, reducing generalizability across different locations. Prediction accuracy may also decline under rapidly changing conditions, such as pandemic-related fluctuations in waste generation. Operational deployment also requires substantial digital infrastructure, sensor integration, technical expertise, and long-term maintenance capacity. Furthermore, the computational demands of AI systems may generate nonnegligible energy costs and contribute indirectly to electronic waste, raising questions about the environmental trade-offs of large-scale implementation.

Therefore, AI should be regarded as an enabling component of integrated waste management systems rather than a standalone solution. Its effectiveness depends on complementary investments in waste segregation, regulatory enforcement, infrastructure development, public participation, and context-specific implementation strategies. A critical priority for future research is the evaluation of cost-effectiveness, scalability, and environmental sustainability of AI-assisted waste management under real-world conditions.

### 10.3. Smart waste bins and Internet of Things integration

The integration of AI with Internet of Things technologies enables the deployment of smart waste bins equipped with sensors that measure fill levels, detect contaminants, and transmit real-time data. AI algorithms evaluate these data streams to prioritize container servicing, prevent overflow, and identify illegal dumping. User-centered smart bin interfaces can provide feedback through mobile applications, promoting proper disposal and recycling behaviors [78].

### 10.4. Resource recovery and waste characterization

AI facilitates the characterization of heterogeneous waste streams through the analysis of spectroscopic, chemical, and visual data. Machine learning models can identify materials suitable for recycling or energy recovery, while AI-driven robotic systems autonomously sort or extract recoverable fractions. These technologies enhance material recovery rates and support the development of advanced waste-to-resource processes [95].

### 10.5. Decision-support systems for policy and planning

AI-based decision-support systems can simulate waste management scenarios, integrate multisource datasets, and evaluate environmental, economic, and social trade-offs. These tools assist policymakers in assessing regulatory options, prioritizing investments, and developing long-term waste management strategies tailored to postpandemic challenges [96]. However, the performance and reliability of these systems can be limited by dataset quality, algorithmic bias, and the substantial computational and energy demands associated with large-scale AI deployment.

## 11. Framework proposal

To enhance conceptual clarity, this review proposes an integrative “Mask Lifecycle–Impact–Response Framework” that links the full trajectory of face mask pollution from production to environmental and policy outcomes. The framework conceptualizes mask-related pollution as a dynamic system involving (i) production and consumption drivers, (ii) environmental release and transport pathways, (iii) physicochemical degradation into MPs and NPs, (iv) ecological and human health impacts, and (v) response mechanisms including waste management systems, regulatory interventions, and technological innovations such as AI-assisted monitoring and sorting (Figure 1).

This integrative model highlights the interconnected nature of environmental contamination and governance challenges, emphasizing that effective mitigation requires coordinated interventions across multiple stages of the mask lifecycle rather than isolated solutions, thereby providing a systems-level perspective that extends beyond conventional single-dimension reviews.

## 12. Conclusion

Face mask pollution has emerged as a multidimensional challenge linking environmental contamination, human health risks, and systemic limitations in current waste management frameworks. The evidence synthesized in this review indicates that mask-derived MPs and associated chemical additives may contribute not only to ecological disruption but also to potential long-term public health burdens, particularly under conditions of chronic exposure and inadequate waste control.

These findings highlight the need for integrated and context-specific strategies that combine regulatory enforcement, sustainable material innovation, and improved waste management practices. Strengthening dedicated collection systems, promoting EPR schemes, and supporting the development of biodegradable or recyclable mask materials are key actionable priorities. In parallel, enhancing public awareness and behavioral interventions will be essential for reducing improper disposal and minimizing environmental leakage.

While emerging technologies such as AI offer opportunities to improve monitoring, sorting, and operational efficiency, their real-world effectiveness remains dependent on data availability, infrastructure capacity, and socioeconomic context. Accordingly, AI should be regarded as a complementary tool within broader system-level waste management strategies rather than a standalone solution.

The available evidence remains uneven in scope and quality, with persistent uncertainties regarding long-term ecological impacts, environmentally realistic toxicological exposure, regional variability in waste management performance, and the scalability of AI-assisted interventions. Inconsistencies in methodological approaches further limit cross-study comparability and hinder the development of standardized risk assessments.

Future research should prioritize standardized monitoring frameworks, environmentally relevant toxicological studies, region-specific evaluations of waste management systems, and real-world validation of technological solutions. Interdisciplinary approaches integrating environmental science, public health, policy analysis, and data science will be essential for developing effective and sustainable responses.

Unlike previous reviews that addressed MP pollution or PPE waste in isolation, this study has adopted a systems-level perspective integrating environmental processes, toxicological evidence, governance challenges, and AI-driven technological solutions. From clinical and public health perspectives, the findings underscore the importance of recognizing face mask pollution as a potential indirect health risk and highlight the need to strengthen the linkage between environmental monitoring and public health systems.

Overall, addressing face mask pollution in the postpandemic era will require coordinated, multisectoral action to ensure sustainable, resilient, and health-protective waste management systems.

## Figures and Tables

**Table 1:** Key assessment strategies for evaluating the environmental, social, and health impacts of face mask litter.

Assessment strategies	Key points	Reference(s)
Ecological risk assessment	Evaluating ecological impacts on ecosystems, biodiversity, and wildlife using modeling and field studies	[37]
Environmental monitoring	Quantifying face mask litter in urban, natural, and aquatic environments through field surveys and monitoring programs	[8,10]
Policy analysis	Reviewing regulations on waste management and litter control to identify gaps and areas for improvement	[20]
Public health surveillance	Monitoring sanitation risks, pathogen exposure, and outbreaks linked to contaminated mask waste	[38]
Social perception surveys	Assessing public attitudes, perceptions, and behaviors toward face mask litter through surveys and social media analysis	[39]

**Table 2:** Reported toxicological effects of common polymers found in face masks.

Polymers	Adverse effects	Reference(s)
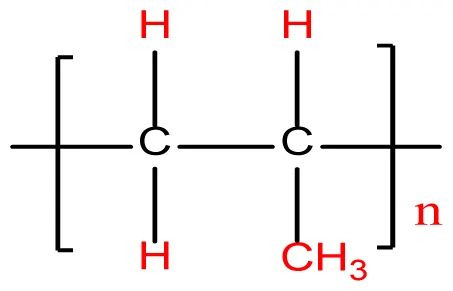 Polypropylene	Pulmonary toxicity, phytotoxicity, carcinogenic potential, gastric injury, inflammatory effects	[47]
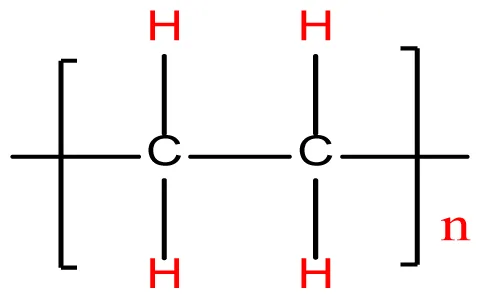 Polyethylene	Carcinogenicity, phytotoxicity, oxidative stress, biochemical and transcriptional alterations, impaired reproduction and development	[40,41]
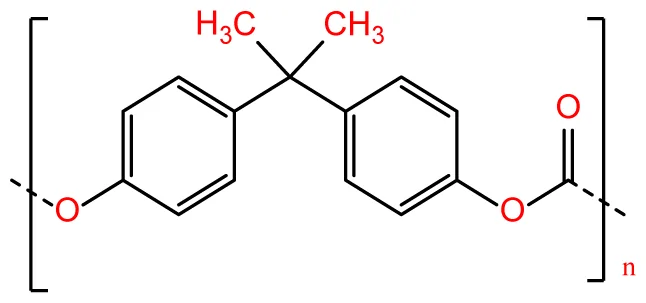 Polycarbonate	Germination inhibition, reduced seedling growth, decreased chlorophyll, increased catalase activity, neurological toxicity	[42]
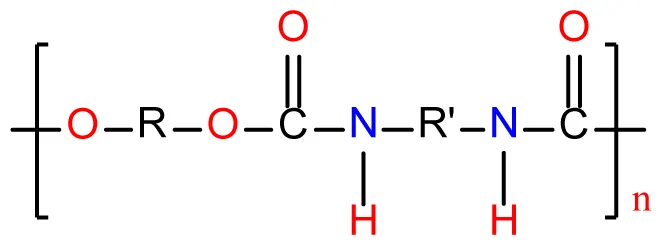 Polyurethane	Tissue damage, carcinogenic and respiratory effects, skin irritation, oxidative stress, endocrine disruption	[43,44]
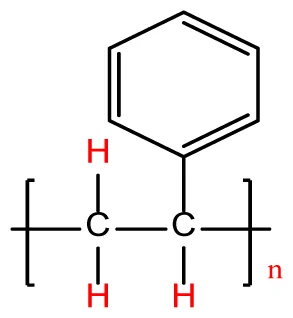 Polystyrene	Oxidative stress, impaired reproduction, enzyme alterations, phytotoxicity, neurotoxicity, immune response disruption	[45]
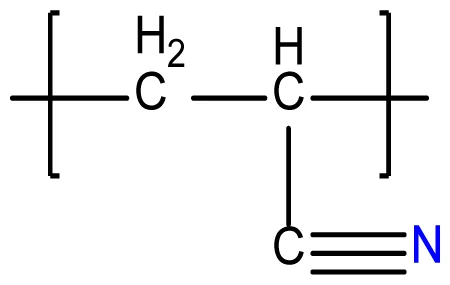 Polyacrylonitrile	Increased heart rate, pericardial edema, altered lipid metabolism in early-life stages	[46]

**Table 3:** Toxic effects of chemical additives associated with face masks.

Chemical additives	Toxic effects	Reference(s)
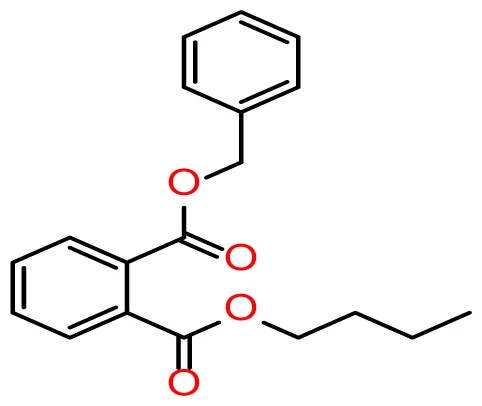 Butyl benzyl phthalate (BBP)	Reproductive toxicity, contact allergy, thyroid disruption, carcinogenic potential	[52]
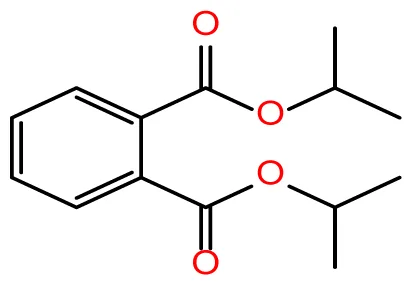 Diisopropyl phthalate (DINP)	Reproductive toxicity, skin/eye irritation, hepatotoxicity, low acute oral toxicity	[54]
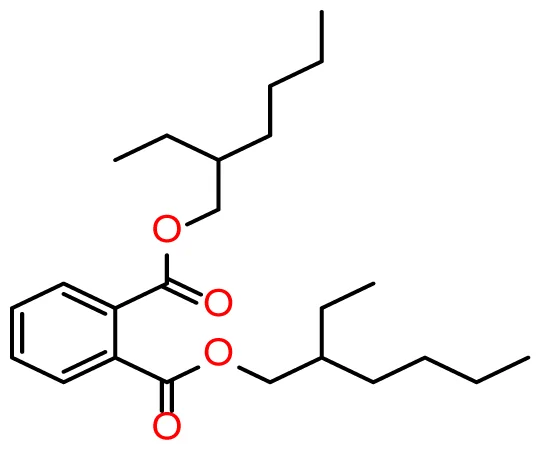 Bis(2-ethylhexyl) phthalate (DEHP)	Androgen disruption, birth defects, germ-cell epigenetic effects, impaired folliculogenesis, neurobehavioral toxicity	[53]
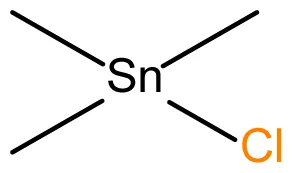 Organotin	Reproductive toxicity, endocrine disruption, cytotoxicity	[49]
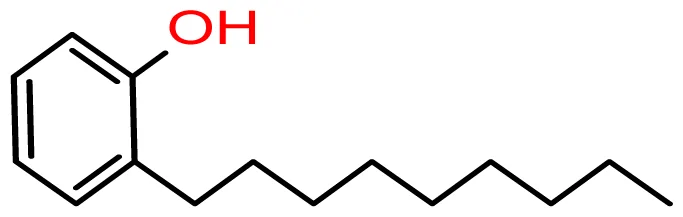 Nonylphenol	Endocrine disruption, impaired reproductive development, altered follicle-stimulating hormone activity, pregnancy complications	[48]
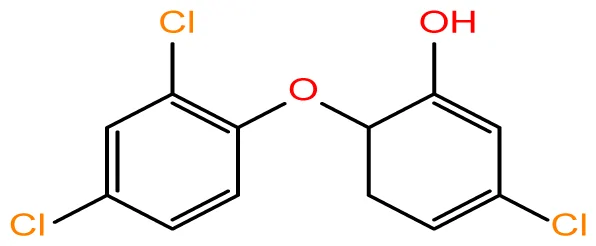 Triclosan	Endocrine disruption, neurotoxicity, genotoxicity, carcinogenicity, phototoxicity, skin sensitization	[50]
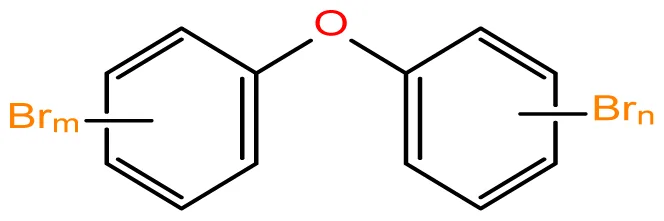 Polybrominated biphenyl ether	Neurotoxicity, immunotoxicity, thyroid disruption, hepatotoxicity, embryotoxicity, metabolic disorders, cancer	[51]

**Table 4:** Mitigation strategies to reduce the toxicological impacts of face mask litter.

Mitigation strategies	Key points	Reference(s)
Public awareness and education	Improve public knowledge on proper disposal and environmental/health risks of discarded masks	[55]
Waste management infrastructure	Strengthen collection, recycling, and disposal systems to prevent environmental leakage of mask waste	[56]
Sustainable materials and design	Encourage biodegradable/recyclable mask materials and ecofriendly product design	[57]
Policy and regulation	Implement regulatory tools (EPR, product stewardship, single-use plastic restrictions)	[58]
Research and monitoring	Conduct monitoring and toxicological studies to inform evidence-based policies	[59]

**Table 5:** Key features of biopolymer-based face masks.

Biopolymer-based face mask	Key points	Reference(s)
Wheat gluten nanofiber membranes	Electrospun gluten–PVA membranes; filtration via electrostatic attraction + physical sieving; ~99% filtration efficiency	[64,65]
Janus membrane filter	Biodegradable PBS microfiber–nanofiber structure coated with chitosan; combines electrostatic capture and fine-pore sieving; removes 98.3% of PM2.5 particles	[66]
PLA electrospun filter	PLA nanofiber filter with 3D mesh printing capability; fiber diameter ~0.83 μm; biodegradable with adequate tensile strength	[67]
Licorice root-based electrospun mask	Electrospun PVA + licorice fibers with high air permeability (pores of 15–30 μm); contains antiviral compounds (glycyrrhizin, 18-glycyrrhetinic acid)	[68]

**Table 6:** Polymer recycling methods and key features.

Recycling methods	Key points	Reference(s)
Aminolysis	Depolymerizes polyurethanes and related polymers through aminolytic cleavage	[78]
Gasification	High-temperature conversion of organic waste to syngas (H_2_, CO), enabling energy or fuel production	[79]
Glycolysis	Chemical depolymerization of polyesters (e.g., PET) using glycols to recover monomers/oligomers	[80]
Hydrolysis	Water-based depolymerization of polymers into constituent monomers under catalytic or thermal conditions	[81]
Hydrogenation	Hydrogen-driven conversion of polymers and organic waste into valuable hydrocarbons or oils	[82]
Pyrolysis	Oxygen-free thermal decomposition producing liquid, gas, and char fractions from plastics and organic waste	[83]

**Table 7:** Key regulatory frameworks governing hazardous waste management in India.

Rules and regulations	Key points	Reference(s)
Environment Protection Act, 1986	India’s central environmental law empowering the government to establish rules on pollution control and waste management	[86]
Hazardous Waste (Management, Handling and Transboundary Movement) Rules, 2016	Regulates generation, storage, transport, treatment, disposal, and cross-border movement of hazardous waste	[87]
Municipal Solid Waste Management Rules, 2016	Establishes standards for collection, segregation, storage, transport, processing, and disposal of municipal solid waste	[88]
Biomedical Waste Management Rules, 2016	Governs segregation, collection, storage, transport, treatment, and disposal of biomedical and infectious waste	[89]
E-waste Management Rules, 2016	Regulates collection, transport, recycling, and disposal of electronic waste, including EPR	[77]

**Table 8:** Comparative overview of AI approaches in waste management and their relevance to face mask pollution, including key applications, strengths, and limitations.

AI approach	Main application	Relevance to face mask waste	Strengths	Limitations
CNN-based computer vision	Object detection and image classification	Detection of discarded masks in streets, beaches, sorting lines	High visual recognition accuracy	Requires large labeled datasets; sensitive to background variability
Deep learning object-detection models	Real-time litter identification	Monitoring mask litter in public spaces and waste facilities	Real-time detection; adaptable to surveillance systems	Computationally intensive; reduced performance under occlusion/degradation
Predictive machine learning	Waste-generation forecasting	Estimating PPE waste volumes and collection demand	Supports planning and route optimization	Dependent on high-quality historical data
AI + Internet of Things smart bins	Fill-level and contamination monitoring	Improved disposal and collection of PPE waste	Real-time operational feedback	High installation and maintenance costs
Decision-support systems	Policy and infrastructure planning	Scenario analysis for postpandemic waste systems	Supports strategic planning	Limited by data quality, model assumptions, and transferability
